# MetaGeno: a chromosome-wise multi-task genomic framework for ischaemic stroke risk prediction

**DOI:** 10.1093/bib/bbaf348

**Published:** 2025-07-18

**Authors:** Yue Yang, Kairui Guo, Yonggang Zhang, Zhen Fang, Hua Lin, Mark Grosser, Deon Venter, Weihai Lu, Mengjia Wu, Dennis Cordato, Guangquan Zhang, Jie Lu

**Affiliations:** Australian Artificial Intelligence Institute, Faculty of Engineering and Information Technology, University of Technology Sydney, Ultimo 2007, New South Wales, Australia; Australian Artificial Intelligence Institute, Faculty of Engineering and Information Technology, University of Technology Sydney, Ultimo 2007, New South Wales, Australia; Australian Artificial Intelligence Institute, Faculty of Engineering and Information Technology, University of Technology Sydney, Ultimo 2007, New South Wales, Australia; Australian Artificial Intelligence Institute, Faculty of Engineering and Information Technology, University of Technology Sydney, Ultimo 2007, New South Wales, Australia; 23Strands, 26-32 Pirrama Road, Pyrmont 2009, New South Wales, Australia; 23Strands, 26-32 Pirrama Road, Pyrmont 2009, New South Wales, Australia; 23Strands, 26-32 Pirrama Road, Pyrmont 2009, New South Wales, Australia; 23Strands, 26-32 Pirrama Road, Pyrmont 2009, New South Wales, Australia; Australian Artificial Intelligence Institute, Faculty of Engineering and Information Technology, University of Technology Sydney, Ultimo 2007, New South Wales, Australia; Department of Neurology and Neurophysiology, Liverpool Hospital, South Western Sydney Local Health District, Liverpool 2170, New South Wales, Australia; Australian Artificial Intelligence Institute, Faculty of Engineering and Information Technology, University of Technology Sydney, Ultimo 2007, New South Wales, Australia; Australian Artificial Intelligence Institute, Faculty of Engineering and Information Technology, University of Technology Sydney, Ultimo 2007, New South Wales, Australia

**Keywords:** genomics and bioinformatics, deep learning, transformers, stroke risk prediction

## Abstract

Current genome-wide association studies provide valuable insights into the genetic basis of ischaemic stroke (IS) risk. However, polygenic risk scores, the most widely used method for genetic risk prediction, have notable limitations due to their linear nature and inability to capture complex, nonlinear interactions among genetic variants. While deep neural networks offer advantages in modeling these complex relationships, the multifactorial nature of IS and the influence of modifiable risk factors present additional challenges for genetic risk prediction. To address these challenges, we propose a Chromosome-wise Multi-task Genomic (MetaGeno) framework that utilizes genetic data from IS and five related diseases. The framework includes a chromosome-based embedding layer to model local and global interactions among adjacent variants, enabling a biologically informed approach. Incorporating multi-disease learning further enhances predictive accuracy by leveraging shared genetic information. Among various sequential models tested, the Transformer demonstrated superior performance, and outperformed other machine learning models and PRS baselines, achieving an AUROC of 0.809 on the UK Biobank dataset. Risk stratification identified a two-fold increased stroke risk (HR, 2.14; 95% CI: 1.81–2.46) in the top 1% risk group, with a nearly five-fold increase in those with modifiable risk factors such as atrial fibrillation and hypertension. Finally, the model was validated on the diverse All of Us dataset (AUROC = 0.764), highlighting ancestry and population differences while demonstrating effective generalization. This study introduces a predictive framework that identifies high-risk individuals and informs targeted prevention strategies, offering potential as a clinical decision-support tool.

## Introduction

Genome-wide association studies (GWAS) have identified numerous genetic loci associated with ischaemic stroke (IS) and its subtypes, leading to the development of methods that use genetic information for IS risk prediction [1–3]. Genetic-based prediction approaches offer advantages over traditional risk assessment tools, such as the Framingham Stroke Risk Profile, which primarily focuses on conventional factors like age, blood pressure, and medical history [4]. By incorporating genetic information, these approaches can identify high-risk individuals who might otherwise be misclassified as low-risk due to the absence of conventional risk factors [5, 6]. Among these genetic-based methods, polygenic risk scores (PRS) are widely employed. PRS models estimate an individual’s genetic risk for IS by calculating a weighted sum of single nucleotide polymorphisms (SNPs) identified in GWAS, where each SNP is weighted by its effect size [7]. This approach has shown potential in stratifying individuals by their stroke risk [8–10]. For instance, Malik *et al*. [8] showed that PRS could effectively stratify individuals by their risk of IS, with individuals in the highest decile of PRS having significantly higher risk compared with those in the lowest decile. Li *et al*. [9], illustrated how PRS can enhance stroke subtyping and improve risk prediction accuracy by incorporating ancestry-specific genetic variants and integrating data from multiple large-scale GWAS. Additionally, Neumann *et al*. [10] applied PRS to predict the risk of IS in a healthy older population, utilizing a comprehensive model that combined genetic risk scores (GRSs) with traditional risk factors, demonstrating the broad applicability of PRS. These studies have collectively highlighted the potential of PRS in improving IS risk prediction. However, the predictive power of PRS can be limited by the model’s linear nature and the lack of consideration for potential nonlinear relationships or epistatic interactions among SNPs, which is crucial for understanding the genetic architecture of complex diseases like IS.

To enhance the performance of PRS, several studies have expanded the phenotypes to include stroke-related diseases [11–13]. For example, O’Sullivan *et al*. [11] combined clinical risk factors and PRS to improve stroke-risk assessment among individuals with atrial fibrillation (AF). Their results showed a significant performance improvement compared with models using genetic data for IS alone. Similarly, Jung *et al*. [12] developed a combined PRS model that utilized risk factors such as hypertension (HT) and type 2 diabetes (T2D). This integrated approach demonstrated enhanced prediction performance, highlighting the benefit of considering multiple related phenotypes in stroke risk prediction. Apart from this, incorporating genotype information with lifestyle factors has also been a research focus. Khera *et al*. [13] combined GRSs with lifestyle factors like diet and physical activity, demonstrating improved predictive accuracy. Likewise, Elliott *et al*. [14] integrated genetic data with smoking and alcohol consumption history, further enhancing the robustness of PRS models. Additionally, incorporating multi-omics data, including transcriptomics and proteomics, has also been shown to provide a more comprehensive risk assessment [15–17]. However, each of these methods has limitations. While incorporating variants from IS-related diseases can enhance risk stratification by leveraging shared genetic information, simply increasing the number of variants without optimizing the model may not significantly improve risk prediction. Instead, it introduces a large number of features, potentially increasing computational complexity and reducing efficiency. For methods combining genetic and lifestyle factors, the linear nature of these models is not fundamentally addressed, nor is the predictive performance of the genetic component significantly enhanced. Lastly, multi-omics approaches often have limited data availability, making them more suitable for model validation rather than primary prediction.

Deep neural networks (DNNs) can model complex interactions between multiple input features [18], making them particularly suited for tasks like genetic risk prediction, where the nonlinear nature of human gene product interactions plays a crucial role [19, 20]. Moreover, DNNs can extract hierarchical features from the genetic data, capturing local and global dependencies among genetic variants [21]. This hierarchical feature extraction is essential for understanding the multifaceted nature of genetic contributions to diseases like IS, hence improving predictive performance. In the biomedical field, DNNs have shown success in various prediction tasks [22–24]. Nonetheless, current DNN methods and PRS models often have limited clinical interpretability because stroke is a multifactorial disease influenced by a wide range of modifiable risk factors (MRFs), including related conditions, lifestyle choices, and environmental exposures. These MRFs, such as HT and AF, can also be analyzed from a genetic perspective to better assess stroke risk. Therefore, clinical risk analysis models should not only enhance genetic risk assessment but also identify optimal strategies for controlling these MRFs. For example, a recent study by Abraham *et al*. [25] developed a meta-PRS approach incorporating 19 GRSs and utilized over 3 million SNPs. This approach achieved a hazard ratio (HR) of 1.26 per standard deviation increase in the meta-PRS (95% CI 1.22–1.31), significantly outperforming most current individual GRSs. However, the reliance on such a large number of features presents several limitations. First, it significantly increases computational complexity and resource requirements, making the model less practical for clinical settings where rapid and efficient risk assessment is needed. More importantly, this approach still adheres to a linear model framework, which does not fully capture the complex, nonlinear interactions between genetic variants, nor does it account for the interplay between these variants and other stroke-related risk factors. Additionally, accurately allocating stroke versus non-stroke labels in long-term risk prediction using genomic data poses a significant challenge in stroke risk prediction, as stroke is often influenced by latent factors that may not manifest as observable symptoms until later stages, potentially leading to the misclassification of individuals as low-risk despite having significant underlying genetic risk. Therefore, there is a need to develop a model that effectively handles a manageable number of features while capturing the nonlinear interactions between genetic variants. Moreover, beyond improved model accuracy, it is more important to understand how MRFs interact with the genetic predispositions of IS. This will enable a more comprehensive risk analysis and provide actionable insights for targeted interventions and personalized stroke prevention strategies.

In this study, we propose a chromoso **Me**-wise Multi-**ta**sk **Geno**mic (MetaGeno) framework for IS risk prediction that considers underlying interactions among multiple MRFs related to IS, including AF, HT, hypercholesterolemia (HCL), T2D, and coronary artery disease (CAD). We introduce a chromosome-based feature extractor designed to capture local and global dependencies among genetic variants within the same chromosome by categorizing SNPs based on their chromosomal locations. This design aligns with the biological principle that genetic variants are more likely to interact when located in the same proximity, such as through linkage disequilibrium (LD) [26, 27]. Moreover, structuring features according to chromosomal organization reflects the inherent architecture of the genome, enabling the model to effectively capture both localized regulatory effects and broader chromosome-level contributions to IS risk. Next, we test several DNN models, including Convolutional Neural Network (CNN) [28], Long Short-Term Memory (LSTM) [29], Gated Recurrent Unit (GRU) [30], Transformer [31], and Temporal Convolutional Network (TCN) [32], each leveraging unique capabilities to capture the intricate relationships between genetic variants across different MRFs. The performance of these models is evaluated using the area under the receiver operating characteristic curve (AUROC) for each MRF prediction, and the best-performing model is compared with baseline methods, such as PRS and traditional machine learning approaches. We further assess the model’s discriminatory power using the concordance index (C-index) and perform cumulative incidence analyses to evaluate its effectiveness in stratifying individuals by IS risk.

## Material and method

This section outlines our study’s methodology, including the dataset description, preprocessing steps, chromosome-wise embedding layers, baseline comparisons, and model architecture.

### Overall design of MetaGeno

**Figure 1:**
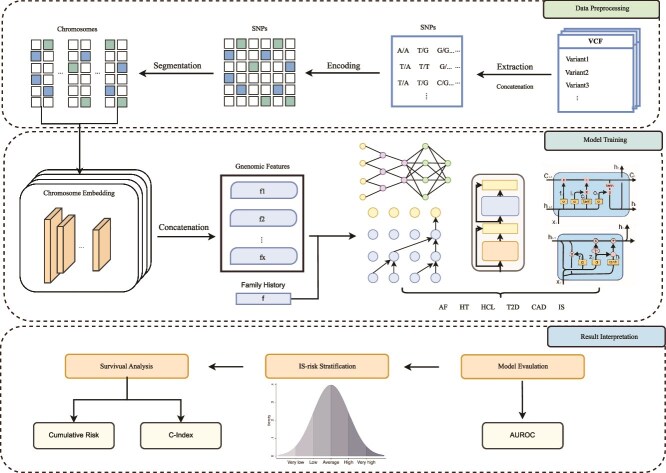
Overall structure of MetaGeno framework for IS risk prediction.

In this study, as shown in Fig. 1, we proposed the MetaGeno framework for predicting the risk of IS by leveraging genetic variants associated with IS and five related MRFs. First, GWAS were selected for each condition, and SNPs were extracted from VCF files. These SNPs were then encoded to represent genetic variants and segmented according to their respective chromosomes. A chromosome-wise embedding layer was utilized for each condition to capture the complex, nonlinear interactions within each chromosome and between adjacent SNPs, enabling the model to recognize key genetic patterns.

Next, we employed a multi-task learning approach to predict IS and five related MRFs: AF, CAD, T2D, HT, and HCL. This multi-task setup utilized the shared genetic information among these conditions, allowing the model to learn from overlapping genetic traits and thereby improve IS prediction accuracy. Various sequential prediction models, including Transformer, LSTM, GRU, CNN, and TCN, were tested to identify the most effective model for this framework.

The performance of each model was evaluated using the AUROC metric, which measures the model’s ability to distinguish between positive and negative classes. We then extracted the output from the final layer of each model and normalized these outputs according to the distribution of the validation population. This enabled us to stratify the predicted IS risk into tertiles, reflecting low, medium, and high-risk groups. For a more refined analysis, we also performed percentile-based stratification to evaluate the risk across finer divisions of the predicted risk distribution. This allowed for a detailed understanding of risk gradations and the model’s discriminative capability. To further investigate the relationship between the MRF and assess the model’s performance in capturing the shared genetic underpinnings, we calculated the C-index for various combinations of MRF. This analysis helped us to quantify the concordance between predicted and observed outcomes across different disease combinations, thereby understanding which genetic overlaps among these conditions could enhance the prediction of IS risk. Next, to validate the identified disease associations and the model’s effectiveness, we evaluated the cumulative incidence of IS by using age as a timeline to generate incidence curves across different MRF combinations and family history. Finally, we validated the model’s performance on an independent dataset to assess its generalizability.

### Datasets and data preparation

The dataset used in this study is derived from the UK Biobank, a large-scale biomedical database and research resource containing genetic, lifestyle, and health information from UK participants [33]. The UK Biobank cohort consists of $\sim $500 000 participants aged 40–69 years who were recruited between 2006 and 2010. To train and test the model, we included 11 584 cases of ischemic stroke and 460 985 control participants without a history of stroke. IS patients were selected based on the source of the ischemic stroke report. Specifically, we included cases reported through hospital admissions, hospital primary diagnoses, hospital secondary diagnoses, stroke-related deaths (either as the primary cause or a contributory factor), and cases where stroke was the sole cause of death, where cases that were solely self-reported were removed. We included all stroke patients with both incident and recurrent strokes, as well as fatal and nonfatal cases. The rationale behind this inclusive approach is that our study aims to identify genetic risk factors for IS, which are relevant regardless of whether the stroke is a first-time event or a recurrence. Transient ischemic attacks were excluded from the analysis as they do not meet the full clinical definition of stroke. We included only participants of European ancestry (EUR) to minimize population stratification and ensure the reliability of our genetic analysis and risk prediction model. Our primary goal was to predict ischemic stroke risk based on an individual’s germline genome, which is inherently static and remains unchanged throughout life. As such, during the selection of samples and the training of our prediction model, we did not incorporate time-dependent variables, such as follow-up time, as these factors are not directly relevant to the genetic predisposition we aim to assess. We employed k-fold cross-validation with $k=5$ to ensure robust model evaluation and minimize variance introduced by a single train-test split. In each fold, the dataset was divided into 80% training and 20% validation. To maintain class balance during training, we applied stratified sampling within each fold, ensuring that the number of positive and negative samples remained equal. Specifically, in each training fold, we randomly selected a subset of healthy individuals, matching the number of positive samples (7500). The remaining samples in each validation fold were used for model testing and analysis.

To further validate the model, we used an independent dataset from the All of Us (AoU) program [34]. The AoU program, an initiative led by the National Institutes of Health in the USA, aimed to enhance healthcare by facilitating precision medicine research through comprehensive data collection, including genetic, environmental, and health information, to define clinical features and outcomes for prediction model development. The AoU program began in May 2018 and recruited individuals aged 18 or older from over 340 recruitment sites across the United States. For this validation, we selected 7936 positive cases of IS and 8000 healthy control participants. The selection criteria mirrored those applied to the UKBB cohort, ensuring consistency in case definition and control exclusion. However, to evaluate the generalizability of our model, we included participants of diverse ancestries in the AoU dataset. Baseline characteristics of the two datasets used in this study are summarized in Table 1.

**Table 1:** Baseline characteristics of the study population. Age is reported as mean $\pm $ SD. Family history of IS is reported as n (%) of the total population

**Characteristic (UKBB)**	**Incident IS** ($\boldsymbol{n = 11\,584}$)	**Healthy controls** ($\boldsymbol{n = 460\,985}$)	**Total** ($\boldsymbol{n = 472\,569}$)
Age, years (mean $\pm $ SD)	62.1 $\pm $ 6.5	56.8 $\pm $ 8.0	57.0 $\pm $ 8.0
Male, n (%)	7011 (60.5)	208 195 (45.2)	215 206 (45.5)
Female, n (%)	4573 (39.5)	252 790 (54.8)	257 363 (54.5)
Family history of IS, n (%)			
No	7754 (66.9)	340 111 (73.8)	347 865 (73.6)
Yes	3830 (33.1)	120 874 (26.2)	124 704 (26.4)
**Characteristic (AoU)**	**Incident IS ($\boldsymbol{n}$ = 7942)**	**Healthy controls ($\boldsymbol{n}$ = 8000)**	**Total ($\boldsymbol{n}$ = 15 942)**
Age, years (mean $\pm $ SD)	70.5 $\pm $ 6.8	68.4 $\pm $ 7.5	69.4 $\pm $ 7.6
Male, n (%)	3577 (45.1)	3205 (40.1)	6782 (42.5)
Female, n (%)	4365 (54.9)	4795 (59.9)	9160 (57.5)
Family history of IS, n (%)			
No	5104 (64.3)	5892 (73.7)	10 996 (69.0)
Yes	2838 (35.7)	2108 (26.3)	4946 (31.0)

The target variable was a set of binary classifications representing the condition of experiencing IS and related phenotypes, including AF, HT, HCL, T2D, and CAD. Each disease was associated with a set of GWAS results obtained from the PGS Catalog [35], including CAD: PGS002262, including 540 SNPs, AF: PGS000035, including 1168 SNPs, HT: PGS003012, including 361 SNPs, T2D: PGS003107, including 995 SNPs, HCL: PGS002274, including 279 SNPs, and IS: PGS000665, including 32 SNPs. The primary objective of this study is to develop an effective framework for IS risk prediction. To avoid data leakage, GWAS datasets were specifically chosen to exclude any data derived from the UK Biobank. The targeted selection of these datasets was guided by their relevance to IS and its major risk factors, the adequate number of SNPs available for robust modeling, their publication in high-quality journals, and their general applicability to populations comparable with the UK Biobank cohort. SNPs identified from these GWAS datasets were used exclusively as input features in our model, independent of GWAS-reported $P$-values or effect sizes, allowing the predictive power to naturally emerge from our chromosome-wise multi-task learning framework. Additionally, our model integrates polygenic risk factors across multiple MRFs, ensuring a more comprehensive risk stratification rather than single-trait genetic prediction.

### Baseline models

This study employed three categories of baseline methods to evaluate the predictive performance of our framework: PRS-based methods, machine learning-based methods, and deep learning-based methods. Detailed model configurations and hyperparameter settings are provided in the appendix.

The first category includes PRS-based methods, which estimate individual disease risk using genetic data. Traditional PRS, a widely used approach in genetic epidemiology, aggregates the effects of multiple genetic variants through a linear model, limiting its ability to capture complex genetic interactions. Therefore, we included two advanced PRS methods: LDpred2 [36] and Lassosum [37]. LDpred2 incorporates LD information to adjust SNP effect sizes, enabling the modeling of genetic dependencies among variants. Lassosum integrates Lasso regression with GWAS data, using $L_{1}$ regularization for efficient feature selection and weight optimization.

The second category includes machine learning-based methods, which model complex and nonlinear relationships in genetic data. These methods include Lasso Regression (LR), Random Forest (RF), Support Vector Machine (SVM), and LightGBM (LGBM). LR applies $L_{1}$ regularization for feature selection, while RF constructs ensemble decision trees to model nonlinear interactions. SVM leverages kernel functions to handle high-dimensional spaces, and LGBM uses gradient boosting to achieve high accuracy in large-scale genetic data analysis. Hyperparameters for these models were optimized using cross-validation to ensure robust performance.

The third category consists of deep learning-based methods, which leverage neural networks to model complex genetic interactions beyond conventional ML approaches. We included two deep learning models, DeepRisk [38] and GenNet [39], to compare with our proposed framework. DeepRisk employs a BiLSTM-based architecture to capture long-range dependencies between SNPs, allowing for the modeling of nonlinear genetic interactions. This enables it to improve upon traditional PRS methods by accounting for complex LD patterns. GenNet, on the other hand, utilizes a neural network architecture that establishes connections between input SNPs and corresponding genes based on gene annotations and further links these genes to relevant biological pathways using pathway annotations to improve interpretability.

### Chromosome-specific embedding layer

Several studies suggest that the spatial organization of the genome plays a critical role in gene regulation and disease susceptibility. Genetic variants located on the same chromosome, especially those in close proximity, tend to exhibit stronger interactions due to LD and co-regulation of gene expression [40, 41]. These interactions contribute to complex phenotypes, including the development of diseases such as IS. Grouping SNPs by chromosome helps preserve these local genetic interactions while enabling the exploration of broader, chromosome-wide effects. Additionally, chromosomal structures align with the biological principle that certain SNPs co-occur within functionally related genomic regions, such as enhancers, promoters, or noncoding regulatory elements [42]. Prior studies have demonstrated the value of structure-aware embedding in modeling complex biological or network data [43], hence this design enables our model to capture both local dependencies and global influences within the same chromosome, enhancing interpretability while addressing the limitations of conventional PRS, which fail to account for nonlinear interactions between SNPs.

Let $\mathcal{D} = \{D_{1}, D_{2}, \ldots , D_{k}\}$ represent the set of diseases considered in this study, where $k$ is the total number of diseases. For each disease $D_{i} \in \mathcal{D}$, we extract relevant SNPs from GWAS and integrate them into a unified feature space, after which they are categorized according to their respective chromosomes. The set of chromosomes is denoted as 


\begin{align*} & \mathcal{C} = \{C_{1}, C_{2}, \ldots, C_{22}\}, \end{align*}

which includes 22 autosomes as sex chromosomes are not included in this analysis.

For a given chromosome $C_{j}$, the set of SNPs located on $C_{j}$ is represented as 


\begin{align*} & \mathbf{s}_{j} = \{s_{j1}, s_{j2}, \ldots, s_{jm}\}, \end{align*}

where $m$ is the total number of SNPs on chromosome $C_{j}$ aggregated across all diseases. Each SNP $s_{jk}$ is encoded as 


\begin{align*} & \mathbf{x}_{jk} \in \{0,1,2\}^{V}, \end{align*}

where $V$ represents the number of possible allele states: homozygous reference (0), heterozygous (1), and homozygous alternative (2).

To capture the shared genetic architecture across related MRFs, we use a single embedding matrix for all diseases. This enables learning common genetic representations for IS and each MRF while preserving chromosome-specific interactions. Embedding all SNPs in a unified space ensures that relevant disease comorbidities information, such as the genetic interplay between IS and its MRFs, is retained for downstream modeling. A single shared embedding matrix is therefore defined as 


\begin{align*} & \mathbf{E} \in \mathbb{R}^{V \times d}, \end{align*}

where $d$ is the embedding dimension. The embedding representation for SNP $s_{jk}$ in chromosome $C_{j}$ is given by 


\begin{align*} & \mathbf{e}_{jk} = \mathbf{E} \mathbf{x}_{jk}. \end{align*}

Thus, the embedded sequence for chromosome $C_{j}$ is represented as 


\begin{align*} & \mathbf{E}(\mathbf{s}_{j}) = \{\mathbf{e}_{j1}, \mathbf{e}_{j2}, \ldots, \mathbf{e}_{jm}\}. \end{align*}

To capture intra-chromosomal dependencies, we use the self-attention mechanism on each chromosome, allowing SNPs within the same chromosome to interact dynamically: 


\begin{align*} & \mathbf{h}_{jk} = \sum_{l=1}^{m} \alpha_{jkl} \mathbf{v}_{jl}, \quad \text{where} \quad \alpha_{jkl} = \frac{\exp\left( \frac{\mathbf{q}_{jk}^\top \mathbf{k}_{jl}}{\sqrt{d}} \right)}{\sum_{l^{\prime}=1}^{m} \exp\left( \frac{\mathbf{q}_{jk}^\top \mathbf{k}_{jl^{\prime}}}{\sqrt{d}} \right)}, \end{align*}

where $\mathbf{h}_{jk}$ represents the final contextualized embedding of SNP $s_{jk}$ on chromosome $C_{j}$, incorporating information from all other SNPs on the same chromosome. The attention weight $\alpha _{jkl}$ quantifies the influence of SNP $s_{jl}$ on $s_{jk}$, with $\mathbf{q}_{jk} = \mathbf{W}_{Q} \mathbf{e}_{jk}$, $\mathbf{k}_{jl} = \mathbf{W}_{K} \mathbf{e}_{jl}$, and $\mathbf{v}_{jl} = \mathbf{W}_{V} \mathbf{e}_{jl}$ representing the query, key, and value embeddings, respectively. Here, $j$ indexes the chromosome, $k$ indexes the SNP receiving attention, and $l$ indexes the SNP contributing information. The projection matrices $\mathbf{W}_{Q}$, $\mathbf{W}_{K}$, and $\mathbf{W}_{V}$ are learnable parameters, and $d$ is the embedding dimension with $\sqrt{d}$ serving as a scaling factor to stabilize training.

To obtain a chromosome-level representation, we apply mean pooling across all SNP embeddings within each chromosome: 


\begin{align*} & h_{j} = \frac{1}{m} \sum_{k=1}^{m} \mathbf{h}_{jk}, \end{align*}

where $h_{j}$ represents the aggregated embedding of chromosome $C_{j}$, summarizing the intra-chromosomal interactions among all SNPs. This attention-based mechanism enables the model to dynamically capture dependencies among SNPs within the same chromosome while reducing dimensionality for downstream analysis.

Once chromosome-level embeddings are obtained, we concatenate embeddings from all chromosomes to form a single chromosome-aware representation: 


\begin{align*} & \mathbf{Z} = \text{concat}(h_{1}, h_{2}, \ldots, h_{22}), \end{align*}

where $\mathbf{Z} \in \mathbb{R}^{22 \times d}$ serves as the final chromosome-aware embedding representation that is dynamically updated throughout the training process via end-to-end optimization through backpropagation, ensuring that both SNP-level and chromosome-level embeddings capture disease-relevant genetic interactions.

### DNN prediction models

We fed the combined embeddings $\mathbf{Z}_{final}$ into various DNN models to predict the risk of IS and its five related phenotypes. Each model leverages unique architectures to capture complex interactions and dependencies within the sequential data.

The 1D CNN model extracts local patterns and hierarchical features from genetic sequences through convolutional layers. Pooling layers reduce dimensionality, and the resulting features are passed to fully connected layers for classification [18].

The LSTM model effectively captures temporal dependencies with memory cell structures that maintain long-range information. Multiple stacked LSTM layers sequentially process inputs, with final hidden states used for classification [44]. GRUs, a simplified variant of LSTMs, reduce computational complexity by combining forget and input gates into a single update gate while retaining similar performance [45].

The transformer model uses self-attention mechanisms to capture global dependencies in genetic data. Its multi-head attention enables the model to jointly attend to information from different representation subspaces, making it particularly effective for long-range interactions [46].

TCNs leverage dilated convolutions to efficiently capture long-range dependencies, maintaining sequence length through input padding [47]. This architecture makes TCNs suitable for modeling potential interactions between genetic variants.

## Results

In this section, we present the performance of our deep learning models compared with baseline methods. We also detail the risk stratification analysis, C-index evaluation for disease combinations, and cumulative risk assessment by age.

### Model selection for genomic chromosome-wise multi-task framework

We evaluated several sequential models to identify the most suitable architecture for the MetaGeno framework to capture the genetic information and interactions in multi-task settings of IS and related MRFs (Table 2). The transformer model consistently outperformed other architectures across all diseases, with an average AUROC of 0.792 and a peak of 0.809 for IS. This strong performance highlights the Transformer’s ability to capture long-range dependencies and leverage inter-disease interactions, which are crucial for understanding polygenic traits like stroke.

**Table 2:** Performance comparison of deep learning models for multi-task classification of IS and related MRFs. Shown are the AUROC scores for the multi-task classification in the validation set of IS and five related MRFs using various deep learning models, including Transformer, LSTM, GRU, CNN, and TCN

**MRFs**	**Transformer**	**LSTM**	**GRU**	**CNN**	**TCN**	**Average**
IS	**0.809**	0.789	0.769	0.776	0.761	**0.781**
AF	**0.800**	0.786	0.762	0.770	0.751	0.774
CAD	**0.790**	0.774	0.757	0.763	0.741	0.765
T2D	**0.768**	0.753	0.735	0.740	0.722	0.744
HT	**0.803**	0.789	0.770	0.777	0.755	0.779
HCL	**0.780**	0.767	0.752	0.758	0.745	0.760
**Average**	**0.792**	0.773	0.758	0.764	0.746	

LSTM and CNN models showed comparable results, with average AUROCs of 0.777 and 0.764, respectively. LSTMs performed well on IS (0.789) and HT (0.789), effectively handling short- to medium-range dependencies. CNNs, traditionally strong in spatial data processing, achieved AUROCs of 0.776 for IS and 0.777 for HT. In contrast, GRU and TCN models exhibited lower performance, with average AUROCs of 0.758 and 0.746, likely due to their limited ability to capture broader genomic interactions.

Performance varied across diseases. IS consistently showed the highest AUROCs, benefiting from its prioritization in the multi-task framework and shared genetic interactions with related conditions. AF and HT also demonstrated strong results (AUROCs of 0.800 and 0.803), reflecting their strong genetic basis. Conversely, CAD, T2D, and HCL had lower AUROCs, particularly with GRU and TCN models, due to the multifactorial nature of these diseases and weaker genetic signals.

Overall, the transformer model’s superiority across diseases underscores its robustness in capturing complex genetic interactions, making it the optimal choice for our framework.

### Capturing local and global dependencies of genetic variants

To assess the ability of our proposed chromosome-wise embedding layer to capture genetic interactions and improve model performance, we conducted a comparison using the same transformer model with different input representations: one using our chromosome-wise embedding layer, another employing a simple one-hot encoding of SNPs, and two additional approaches with a global SNP embedding where all SNPs share the same embedding space, and another adopting an independent SNP embedding where each SNP has its own embedding layer.

**Figure 2:**
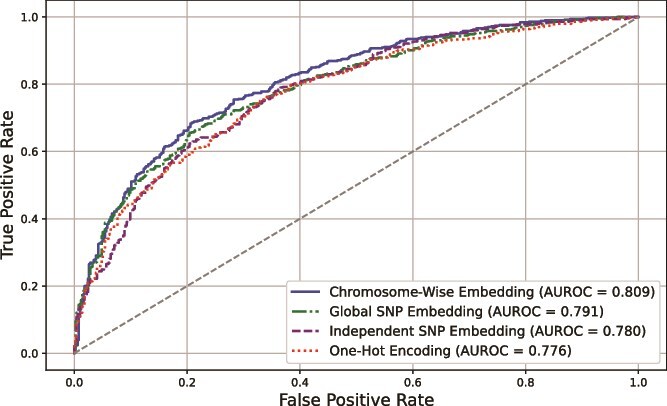
**AUROC comparison between different SNP embedding strategies in the transformer model.** Shown are the AUROC curves for the same transformer model using different input representations. The blue curve represents the chromosome-wise embedding layer, achieving the highest AUROC of 0.809. The green curve corresponds to the global SNP embedding, resulting in an AUROC of 0.791. The purple curve represents the independent SNP embedding, yielding an AUROC of 0.78. The red curve denotes the one-hot encoding baseline which achieved an AUROC of 0.776.

As shown in Fig. 2, the AUROC curves illustrate that the chromosome-wise embedding layer (blue curve) achieved the highest AUROC of 0.809, outperforming the global SNP embedding (green curve, AUROC = 0.791), the independent SNP embedding (purple curve, AUROC = 0.780), and the one-hot encoding (red dotted curve, AUROC = 0.776). These results suggest that while deep learning-based embedding strategies generally improve over traditional one-hot encoding, incorporating the hierarchical genetic structure into the embeddings, particularly by grouping SNPs at the chromosome level, could further enhance model performance.

The performance gap between chromosome-wise and global SNP embeddings highlights the importance of maintaining structural genomic information, as global embedding loses the local dependencies between SNPs within the same chromosome. Additionally, the lower AUROC of the independent SNP embedding method suggests that failing to capture any SNP–SNP interactions weakens the predictive power, despite allowing individual SNPs to be represented in a lower dimensional space. These findings support our hypothesis that incorporating both local and global genetic dependencies through chromosome-wise embeddings contributes to better disease risk prediction.

### Comparison of IS prediction performance with baselines

Table 3 compares the performance of the proposed MetaGeno framework with several baseline models for IS prediction. MetaGeno achieved the highest AUROC score of 0.809, demonstrating its ability to capture complex, nonlinear interactions within chromosomes and leverage shared genetic information across related diseases. This was followed by LGBM (AUROC = 0.778) and RF (AUROC = 0.761), which performed well but lacked the ability to utilize multi-task learning and cross-disease interactions, limiting their effectiveness.

**Table 3:** Performance metrics for the proposed MetaGeno framework against baselines. Shown are the AUROC, F1 Score, Precision, and Recall metrics comparing the performance of the MetaGeno framework with the transformer model against traditional baselines, including PRS, LDpred, Lassosum, LR, RF, SVM, and LGBM for the prediction of IS

**Method**	**AUROC (95% CI)**	**F1 Score**	**Precision**	**Recall**
**MetaGeno (Ours)**	0.809 (0.800–0.816)	0.790	0.780	0.778
LDpred	0.690 (0.680–0.700)	0.675	0.680	0.670
Lassosum	0.670 (0.660–0.680)	0.655	0.660	0.650
PRS	0.650 (0.640–0.660)	0.635	0.640	0.630
DeepRisk	0.792 (0.784–0.800)	0.778	0.780	0.773
GenNet	0.787 (0.779–0.795)	0.774	0.775	0.770
LR	0.734 (0.726–0.745)	0.720	0.730	0.712
RF	0.761 (0.753–0.772)	0.755	0.760	0.750
SVM	0.746 (0.738–0.752)	0.736	0.745	0.726
LGBM	0.778 (0.770–0.789)	0.770	0.778	0.765

The traditional PRS model showed lower performance (AUROC = 0.650, F1 Score = 0.635), reflecting its inability to capture nonlinear interactions or include non-genetic factors. LDpred (AUROC = 0.690, F1 Score = 0.675) and Lassosum (AUROC = 0.670, F1 Score = 0.655) improved upon PRS by leveraging LD information and Lasso-based regularization, but their predictive power remained limited. LR and SVM demonstrated moderate performance (AUROC = 0.734 to 0.746), handling linear and simple nonlinear patterns but struggling with high-dimensional genomic interactions.

Finally, the two deep learning-based methods, DeepRisk and GenNet, achieved higher performance than traditional ML models by effectively capturing complex nonlinear genetic interactions. DeepRisk (AUROC = 0.792) used BiLSTM architectures to model intricate SNP dependencies, while GenNet (AUROC = 0.787) integrated biologically informed neural network structures by grouping SNPs according to their corresponding genes, improving model interpretability. These models demonstrate the potential of deep learning in genomic risk prediction beyond traditional PRS approaches. However, their performance may be constrained by the lack of interaction modeling across multiple MRFs, which share heritability information crucial to IS risk, and the insufficient representation of cross-chromosomal effects, which are essential for capturing long-range genetic dependencies.

### IS risk stratification by MetaGeno framework

**Figure 3:**
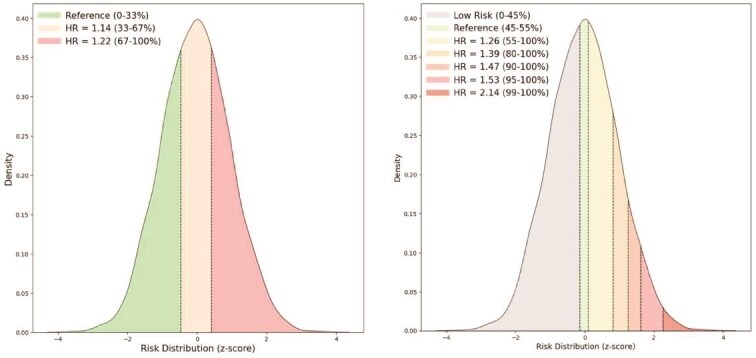
The MetaGeno framework identifies individuals at different risk levels of IS. Shown is the distribution of the risk distributions for IS in the validation set and their corresponding HRs. The left panel displays the HRs for the top risk bins stratified by tertiles: Reference (0%–33%), HR = 1.14 (33%–67%), and HR = 1.22 (67%–100%), with the lowest tertile serving as the reference group. The right panel illustrates a more detailed stratification of risk scores across multiple percentiles: Low Risk (0%–45%), Reference (45%–55%), HR = 1.26 (55%–100%), HR = 1.39 (80%–100%), HR = 1.47 (90%–100%), HR = 1.53 (95%–100%), and HR = 2.14 (99%–100%). These distributions reflect the varying levels of IS risk across different groups.

The MetaGeno framework successfully stratifies individuals into distinct risk categories for IS by employing a multi-level HR analysis based on the putout from the final layer of the model. Figure 3 shows two panels illustrating the risk distributions and their corresponding HRs for IS in the validation set.

The HRs are first calculated and presented for different bins stratified by tertiles: HR = 1.14 (95% CI: 1.07–1.20) for the second tertile (33%–67%), and HR = 1.22 (95% CI: 1.14–1.29) for the highest tertile (67%–100%), using the lowest tertile (0%–33%) as the reference group. This stratification demonstrates a clear gradation of stroke risk as the risk score increases. The HRs indicate that individuals in the highest tertile have a 22% higher risk of stroke compared with the reference group, while those in the middle tertile exhibit a 14% higher risk.

Furthermore, a more detailed stratification of risk scores across multiple percentiles in the higher IS risk region is provided: HR = 1.26 (95% CI: 1.18–1.33) for the 55%–100% group, HR = 1.39 (95% CI: 1.26–1.51) for the 80%–100% group, HR = 1.47 (95% CI: 1.31–1.63) for the 90%–100% group, HR = 1.53 (95% CI: 1.36–1.71) for the 95%–100% group, and HR = 2.14 (95% CI: 1.81–2.46) for the 99%–100% group. This more specific stratification shows that individuals in the top 1% have more than double the risk of stroke compared with those in the reference group. The increasing HRs across percentiles confirm that the MetaGeno framework can differentiate individuals with varying degrees of IS risk, with statistically significant differences between groups as indicated by their respective confidence intervals.

### IS prediction of different combinations of MRFs

**Figure 4:**
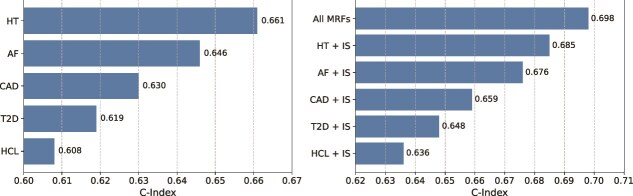
C-Index of Different MRF combinations for IS Prediction. The left panel displays the C-index values for predicting IS using individual GWAS of each related MRF: HT, AF, CAD, T2D, and HCL. The right panel illustrates the C-index values for various combinations of these conditions’ GWAS data with IS. The ”All MRFs” combination represents the integration of GWAS data for all related MRF with IS, yielding the highest C-index.

To validate the effectiveness of the MetaGeno framework in identifying the genetic risk of IS, we assessed its predictive performance using the C-index, a metric that measures a model’s ability to distinguish between different risk levels. By testing the C-index for each related MRF individually, we aimed to determine how much each MRF contributes to the overall risk prediction for IS within a multi-task learning setting. Figure 4 shows that HT achieved the highest C-index of 0.661, followed by AF with a C-index of 0.646. These findings suggest that HT and AF have a strong genetic association with IS, likely due to shared pathophysiological mechanisms contributing to both conditions. In contrast, diseases like CAD, T2D, and HCL had lower C-index values of 0.630, 0.619, and 0.608, respectively, reflecting these conditions’ more complex, multifactorial nature where genetic factors alone are less predictive for the risk of IS.

Subsequently, we evaluated the performance of the MetaGeno framework when combining IS with each related disease. The results demonstrated that integrating multiple diseases significantly enhances predictive accuracy. This suggests that integrating direct and indirect genetic influences across related conditions can capture complex disease interactions, providing a more comprehensive understanding of IS risk. Among the individual combinations, the model combining HT and IS achieved a C-index of 0.685, while AF and IS had a C-index of 0.676, further validating the relevance of these conditions in the context of IS risk prediction. The combinations of CAD + IS, T2D + IS, and HCL + IS also showed improved performance with C-index values of 0.659, 0.648, and 0.636, respectively. Finally, combining all MRFs achieved the highest C-index of 0.698.

### Risk stratification based on genetic factors and family history across age subgroups

**Figure 5:**
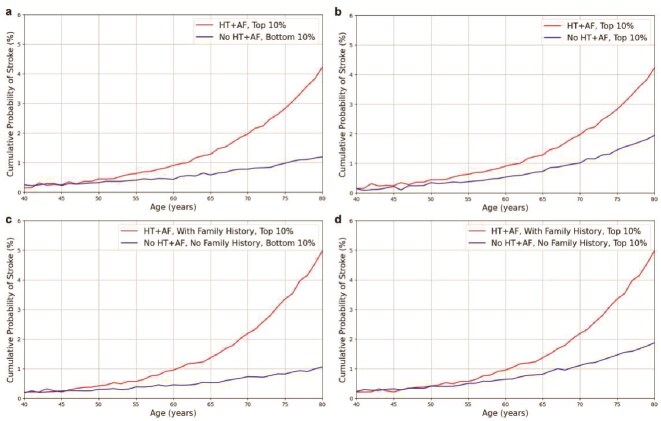
Cumulative probability of stroke by risk subgroups stratified by HT, AF, and family history, across age. Shown are the cumulative probability of stroke across various risk subgroups stratified by HT, AF, and family history, presented as Kaplan–Meier curves across different age groups. The top 10% and bottom 10% genetic risk groups were defined based on the predicted risk scores obtained from the MetaGeno framework. (a) shows the cumulative stroke risk for individuals with HT and AF in the top 10% genetic risk group compared with those without HT and AF in the bottom 10% genetic risk group. (b) compares the stroke risk between individuals with both HT and AF and a family history of stroke in the top 10% genetic risk group versus those without HT and AF and no family history in the bottom 10% genetic risk group. (c) depicts the stroke risk for individuals with HT and AF in the top 10% genetic risk group compared with those without HT and AF within the same top 10% genetic risk group. (d) presents the stroke risk for individuals with both HT and AF and a family history of stroke in the top 10% genetic risk group against those without HT and AF and no family history within the same top 10% genetic risk group.

To further explore the combined effects of HT and AF with IS, given their high correlation observed in the previous sections, we conducted a series of analyses to examine the cumulative probability of IS across multiple risk subgroups stratified by HT, AF, and family history of stroke. The results are shown in Fig. 5, illustrating how stroke risk accumulates across different subgroups as individuals age. For this analysis, we stratified individuals based on their predicted IS risk scores, selecting the top 10% of individuals with the highest predicted risk and the bottom 10% with the lowest predicted risk.

We first assessed the effect of HT and AF in the overall population. For individuals in the top 10% risk group with both HT and AF, the cumulative risk of IS by age 80 reached 4.0% (95% CI: 3.7%–4.4%) (Fig. 5a). In contrast, the cumulative stroke probability was significantly lower for individuals in the bottom 10% group without HT and AF, around 1.2% (95% CI: 1.0%–1.3%), indicating a more than three-fold increase. Next, we compared the risk within the top 10% group; the cumulative stroke probability by age 80 for those without HT and AF increased to 1.9% (95% CI: 1.6%–2.2%) (Fig. 5b), suggesting more than a two-fold increase in stroke risk for those with HT and AF, even within the same high-risk percentile. We then analyzed the impact of having a family history of IS on stroke risk among individuals in the risk distribution. Among individuals in the top 10% with both HT and AF who also had a family history of IS, the cumulative probability of stroke by age 80 reached 4.9% (95% CI: 4.3%–5.1%) (Fig. 5c). Meanwhile, those without HT and AF and family history in the bottom 10% group had the lowest cumulative stroke risk of around 1.0% (95% CI: 0.9%–1.2%), showing a nearly five-fold increase in risk for individuals with both conditions and family history. Finally, for individuals in the bottom 10% risk group and without all three factors (HT+AF, family history), cumulative stroke probability was around 1.8% (95% CI: 1.6%–2.1%) (Fig. 5d). This comparison highlights nearly a three-fold increase in stroke risk for high-risk individuals with all three high-risk characteristics.

These findings underscore the importance of considering multiple interacting risk factors, including HT, AF, and family history, as these risk factors substantially elevate the cumulative risk of stroke, particularly for those within the highest risk groups, with significant increases in risk observed across all comparisons.

### Integration of chromosome-wise attention scores for IS prediction

**Figure 6:**
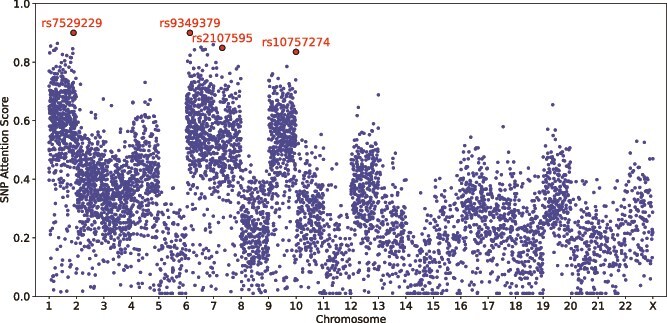
Chromosome-wise attention score distribution. The figure illustrates the normalized attention scores assigned to each chromosome by the transformer model within the MetaGeno framework. Attention scores were computed by aggregating SNP-level attention values within each chromosome and normalizing them to a scale of 0 to 1.

To further understand the contribution of different chromosomes in IS risk prediction, we analyzed the attention scores derived from our transformer-based MetaGeno model. Specifically, we analyzed SNP-level importance scores derived from the attention weights of our transformer model. By aggregating and normalizing the attention assigned to individual SNPs within high-ranked chromosomes, we identified key variants contributing to IS prediction. Figure 6 presents the chromosome-wise attention distribution, where each chromosome’s importance was computed by summing the attention scores across all SNPs within that chromosome and normalizing them to a 0–1 scale. As shown, chromosomes 1, 4, 6, 7, 9, and 12 exhibit higher attention scores, while others contribute to varying degrees.

From a biological perspective, these results align well with-known genetic contributors to IS and its MRFs. Chromosome 1 includes key IS-associated variants such as *rs7529229*, which has been strongly linked to CAD and AF. Chromosome 4 contains the *ZFHX3* gene, which is also associated with AF, further reinforcing its role in IS susceptibility. Chromosome 6 is enriched in genes related to the immune system and inflammation, *rs9349379* within the *PHACTR1* gene is located here and has been linked to arterial function and vascular integrity. Similarly, *rs2107595* in Chromosome 7 has been highly ranked, consistent with prior studies linking this gene to CAD and AF. Chromosome 9 contains another key variant, *rs10757274*, a well-established locus for CAD, associated with both IS and AF. Chromosome 12 contains the *SH2B3* gene, associated with HT and other cardiovascular diseases.

Conversely, chromosomes with lower attention scores, such as Chromosomes 5, 14, and 15, may still contain IS-associated SNPs while their contribution to the model is relatively weaker compared with highly ranked chromosomes. Specifically, Chromosome 5 is largely implicated in immune-related and developmental disorders, with limited direct links to IS and its related MRFs. Chromosomes 14 and 15 have been associated with neurodevelopmental and metabolic syndromes but lack significant cardiovascular risk loci. This observation supports our model’s prioritization of chromosomes with well-established genetic links to IS and its MRFs. These SNPs reflect distinct but interconnected mechanisms contributing to IS risk. Variants in *ZFHX3* influence cardiac electrical activity, predisposing individuals to AF and subsequent cardioembolic stroke. The identification of SNPs in *PHACTR1* suggests an important role in arterial wall remodeling and blood pressure regulation. Additionally, *SH2B3* encodes the lymphocyte adaptor protein LNK, which negatively regulates cytokine signaling and immune cell proliferation. Variants in *SH2B3* have been associated with increased inflammation and HT, further reinforcing the role of immune dysregulation and elevated blood pressure in IS pathogenesis. Collectively, these findings validate our model’s ability to capture functionally relevant genetic interactions and highlight the interplay between AF, HT, and other MRFs in IS risk.

### Model validation using the AoU dataset

**Figure 7:**
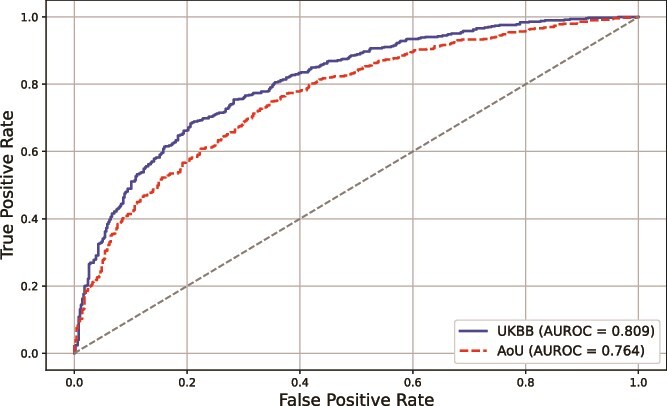
ROC curves for stroke risk prediction on UKBB and AoU datasets. ROC curves comparing the performance of the model on the UK Biobank dataset (AUROC = 0.809) and the AoU validation dataset (AUROC = 0.764).

To further evaluate the robustness and generalizability of our model, we validated its performance on an independent dataset from the AoU program. The ROC curves in Fig. 7 compare the performance of the model on the UK Biobank dataset (training/validation data) and the AoU dataset (external validation). For the AoU validation dataset, the model achieved an AUROC of 0.762, indicating satisfactory generalization to a completely independent cohort with varying demographic and clinical characteristics.

The slightly lower AUROC on the AoU dataset than the UKBB dataset (AUROC = 0.811) may primarily be attributed to differences in ancestry composition and population distribution between the two cohorts. The UK Biobank dataset predominantly consists of individuals of European ancestry, whereas the AoU dataset represents a more diverse population with varying genetic backgrounds. Such differences can introduce additional variability, impacting model performance when applied to a heterogeneous cohort. Despite these challenges, the model achieved an AUROC of 0.762 on the AoU dataset, demonstrating its ability to generalize effectively to completely independent data.

## Discussion

In this study, we proposed a MetaGeno framework for predicting the risk of IS by integrating genetic data from IS and five related MRFs (AF, HT, HCL, T2D, and CAD). Using the proposed chromosome-based embedding layer, we aimed to capture both local and global genetic interactions within the same chromosome. To identify the most effective model for our framework, we explored various deep learning models, including Transformer, LSTM, GRU, CNN, and TCN. The results demonstrated that the transformer model, incorporated into the MetaGeno framework, achieved the highest predictive performance with an AUROC of 0.811, outperforming PRS and machine learning methods. Additionally, the MetaGeno framework effectively stratified individuals into different risk groups. For example, when stratifying by tertiles, individuals in the highest risk group (67%–100%) showed a 1.23-fold increase in IS risk compared with the reference group (0%–33%). The more detailed risk stratification, which divided the population into multiple percentiles, identified individuals in the top 1% (99%–100%) had over a 2.13-fold increased risk of IS compared with the reference group (45%–55%). This demonstrates that the MetaGeno framework can identify individuals at significantly varying risk levels, with the highest risk groups showing substantially elevated stroke risk.

The C-index analysis further highlighted the effectiveness of the MetaGeno framework in utilizing multi-task learning to incorporate information from multiple MRFs. Combining all related diseases yielded the highest C-index of 0.701, suggesting that incorporating genetic data from all these conditions provides a comprehensive understanding of IS risk. Notably, the combination of HT and AF with IS yielded a C-index of 0.685 and 0.677, further supporting the close genetic association of these conditions with IS. To validate the potential relevance of HT and AF in predicting IS risk, we conducted additional survival analyses focusing on these two MRFs. The results indicated that individuals with both HT and AF in the top 10% risk group exhibited more than a two-fold increase in IS risk compared with those without these conditions (4.1% and 1.9%). More importantly, when family history was also considered, individuals with both HT and AF and positive family history in the top 10% risk group had an even greater cumulative stroke risk of 4.9% by age 80. This represents nearly a five-fold increase compared with those without these conditions in the bottom risk group, with a risk of $\sim $1.0%, further underscoring the impact of combining IS-related risk factors and family history on effectively stratifying individuals by their risk levels.

When comparing our findings with previous studies, Marston *et al*. [48] observed that incorporating data from cardiovascular clinical trials enhanced the prediction of IS risk, with HRs of 1.15 (95% CI: 0.98–1.36) for the middle genetic risk tertile and 1.24 (95% CI: 1.05–1.45) for the high genetic risk tertile. Similarly, research by O’Sullivan *et al*. [11] showed improved stroke prediction accuracy among AF patients by using over 500 000 SNPs. Their comprehensive GRS notably increased the net reclassification index (2.3% [95% CI: 1.3%–3.0%]) and improved predictive accuracy ($\chi ^{2} P = 0.002$), achieving a C-index of 0.63 (95% CI: 0.61–0.65). Moreover, Abraham *et al*. [25] developed a meta-PRS strategy that combines 19 GRSs for IS-related SNPs, encompassing over 3 million SNPs, resulting in an HR of 1.26 (95% CI: 1.22–1.31) per standard deviation, which outperformed many existing individual GRS methods. While the results from these studies may appear comparable, our approach offers distinct advancements that address the underlying limitations of their methodologies. These methods often depend on a vast number of SNPs—ranging from 500 000 to over 3 million, which, although potentially improving predictive power, do not fundamentally resolve the nonlinearity issues inherent in traditional PRS approaches. Furthermore, including such extensive feature sets makes their application in DNNs less feasible due to computational constraints and the risk of overfitting. In contrast, our MetaGeno framework achieves similar or improved predictive accuracy using substantially fewer variants, mitigating the limitations of high-dimensional data through a chromosome-based embedding layer that effectively captures local and global nonlinear interactions among genetic variants. Furthermore, by incorporating related diseases through a multitask learning approach, our model leverages shared genetic information, enhancing predictive performance while maintaining a more manageable feature set. Thus, our framework provides a more streamlined, scalable, and clinically applicable solution compared with conventional methods that rely on extensive genomic data and linear PRS models.

Clinically, current stroke risk assessment methods largely emphasize phenotypic risk factors and lifestyle characteristics, such as blood pressure, glucose, and cholesterol levels, even though genetic factors contribute to $\sim $37.9% of IS heritability. This focus can lead to an underestimation of risk in genetically predisposed individuals who appear to be at low risk based on clinical factors alone. Our model bridges this gap by integrating genetic data to offer a more comprehensive risk assessment, allowing for the identification of high-risk individuals who may be overlooked by traditional methods. While family history highlights a strong connection with the genetic factors we previously focused on, this framework also demonstrates that managing other MRFs can significantly reduce the risk of developing IS. Additionally, providing numerical evidence from a large population study for clinically actionable measures could assist clinicians in providing prevention strategies, early intervention, and targeted lifestyle modifications. This would help manage the likelihood of developing IS proactively, hence promoting more efficient healthcare resource allocation by offering clinicians critical insights into a patient’s genetic predisposition to stroke, along with a timeline for risk, supporting more informed decisions regarding preventive medication and lifestyle modifications.

Despite the enhanced predictive accuracy achieved by employing a chromosome-wise embedding layer to capture nonlinear interactions among genetic variants and identifying the impact of AF and HT on stroke risk across different subgroups, there remains room for improvement. Notably, imaging data play a critical role in clinical settings, particularly in the diagnosis, prognosis, and rehabilitation of stroke patients. Imaging biomarkers, such as MRI findings in chronic small vessel disease or intracranial atherosclerosis, are essential for understanding the complete scope of stroke pathology and patient outcomes. Future studies should aim to incorporate imaging data, along with clinical and biomarker information, to explore the interactions and relationships between genetic factors and imaging phenotypes. This multimodal approach would provide a more comprehensive assessment of IS risk and enable personalized treatment plans considering both genetic predisposition and clinical imaging findings. Furthermore, such integration could deepen our understanding of the mechanisms underlying IS, leading to more effective strategies for stroke prevention and outcome analysis.

**Key Points**

- This study proposed a genomic variant-based model integrating five modifiable risk factors (AF, HT, HCL, T2D, CAD) and considering disease interactions for IS risk prediction.
- A biologically informed chromosome-based embedding layer was designed to capture complex interactions between variants on the same chromosome, outperforming one-hot encoding.
- We compared several sequential models, with the transformer model showing the best performance and outperforming baseline methods, including traditional and conventional PRS models.
- Risk stratification analysis identified a 1.23-fold risk increase in the top 67%–100% tertile and a 2.13-fold increase in the top 1%, with nearly five-fold higher risk in individuals influenced by AF, HT, and family history compared with the lowest risk group.

## Supplementary Material

Multi_task_Chromosome_Embedding_Appendix_bbaf348

## Data Availability

The implementation of the MetaGeno framework is publicly available at https://github.com/cireyy/Genomic-Transformer/tree/main.
