# Supplementary material for: MetaGeno: a chromosome-wise multi-task genomic framework for ischaemic stroke risk prediction
Source: Brief Bioinform. 2025 Jul 18;26(4):bbaf348. doi: 10.1093/bib/bbaf348 (PMC12271575; doi:10.1093/bib/bbaf348)
Supplement: Multi_task_Chromosome_Embedding_Appendix_bbaf348 [file multi_task_chromosome_embedding_appendix_bbaf348.pdf]

Appendix

Baseline Models and Hyperparameter Settings

This section describes the software frameworks, model architectures, and hyperparameter settings used in this study.

Software and Frameworks

All models were implemented using **PyTorch** (version 2.1) for deep learning models and **Scikit-learn** (version 1.3.1) and **LightGBM** (version 4.0) for traditional machine learning baselines.

Feature Transformation in Baseline Comparisons

To ensure a fair comparison between our proposed framework and baseline models, we applied different feature transformation strategies depending on the type of model. For deep learning models, SNP data were first mapped to a shared embedding space using our chromosome-wise embedding layer before being processed according to each architecture's specific mechanism. Transformer-based models utilized self-attention to capture dependencies among SNPs within the same chromosome. In contrast, recurrent models such as LSTM and GRU directly received the embedded SNP sequences as sequential input to model temporal dependencies. The entire feature transformation process, including the embedding layer, was trained end-to-end with the model through backpropagation. In contrast, traditional machine learning models such as RF, SVM, LightGBM, and LR were trained directly on raw SNP genotype data, encoded as 0, 1, or 2, without the chromosome-wise embedding transformation.

Machine Learning Baselines

We evaluated our proposed MetaGeno framework against several traditional machine learning models, including LR, RF, SVM, and LGBM. The key hyperparameters for these models were optimized using grid search and cross-validation:

- **LR**: Regularization parameter $C \in \{0.01, 0.1, 1, 10\}$ with both L1 and L2 regularization tested. Features were standardized using *StandardScaler* to improve convergence.
- **RF**: Number of trees $n\_estimators \in \{100, 300, 500\}$, minimum samples per split $min\_samples\_split \in \{2, 5, 10\}$.
- **SVM**: Kernel $\in$ {linear, rbf}, regularization parameter $C \in \{0.01, 0.1, 1, 10\}$. Features were standardized using *StandardScaler* before training.
- **LGBM**: Learning rate $\eta \in \{0.01, 0.05, 0.1\}$, number of boosting rounds $n\_estimators \in \{200, 500, 1000\}$, maximum tree depth $max\_depth \in \{5, 7, 10\}$, and number of leaves $num\_leaves \in \{31, 63, 127\}$ to better handle high-dimensional feature space.

Deep Learning Architectures

We experimented with multiple deep learning architectures, including 1D CNNs, LSTM networks, GRUs, TCNs, and Transformers. All deep learning models were trained using the Adam optimizer with an initial learning rate of 0.001. Batch sizes were optimized in the range $\{64, 128, 256\}$ based on computational efficiency and convergence stability. Models were trained for up to 100 epochs with early stopping (*patience* = 10) based on validation loss. The BCEWithLogitsLoss function was used for classification. The architectural configurations and hyperparameter settings are as follows:

- **1D CNN**: Three convolutional layers with kernel sizes $\{3, 5, 7\}$, activation function ReLU, followed by max pooling layers with pooling size 2. A fully connected layer maps the extracted features to the final classification layer. Batch normalization and dropout (0.3) were applied after each convolutional layer to stabilize training.
- **LSTM**: Two stacked LSTM layers with hidden units $\in \{128, 256\}$, dropout rate of 0.2, and a final dense layer for classification. A bidirectional LSTM variant was tested and showed slight improvement, so it was adopted in the final model.
- **GRU**: Two stacked GRU layers with hidden units $\in \{128, 256\}$, dropout rate of 0.2, and a fully connected classification layer.
- **Transformer**: Two encoder layers, each with eight attention heads, hidden dimension $d_{model} = 512$, feedforward dimension $d_{ff} = 2048$, and ReLU activation. The input embeddings include positional encoding to retain sequence order information. Layer normalization and dropout (0.1) were applied.
- **TCN**: Four convolutional layers with dilation rates $\{1, 2, 4, 8\}$, kernel size 5, and ReLU activation. Residual connections and dropout (0.3) were used to enhance training stability.

Statistical Analysis

Cox Proportional Hazards Model

To assess the association between genetic risk factors and IS incidence, we employed the Cox proportional hazards model, a widely used method in survival analysis for estimating the effect of covariates on time-to-event outcomes. We used age as the time variable, measuring the time from birth until the occurrence of IS or censoring.

The Cox proportional hazards model is defined as:

$$h(t|X) = h_0(t) \exp\left(\sum_{i=1}^{p} \beta_i X_i\right), \quad (1)$$

where $h(t|X)$ represents the hazard function at time t given covariates X, $h_0(t)$ is the baseline hazard function, β_i are the regression coefficients, and X_i are the predictor variables, including genetic risk scores, family history, and clinical risk factors.

The hazard ratio (HR) is a key measure in survival analysis that quantifies the effect of a given risk factor on the likelihood of an event. In the Cox model, the HR for a predictor variable X_i is given by:

$$HR = e^{\beta_i} \quad (2)$$

where β_i is the estimated coefficient for X_i. An HR greater than 1 indicates an increased risk of IS associated with the predictor, while an HR less than 1 suggests a protective effect. For example, an HR of 1.5 implies that individuals with a

specific risk factor have a 50% higher hazard of IS compared to those without the risk factor. Conversely, an HR of 0.8 suggests a 20% lower risk.

We verified the proportional hazards assumption using Schoenfeld residuals. HR and their 95% confidence intervals were computed to quantify the effect of different risk factors on IS incidence. The analysis was adjusted for age, sex, genetic ancestry (by principal components 1–5), and clinical comorbidities, including hypertension, atrial fibrillation, coronary artery disease, type 2 diabetes, and hypercholesterolemia, which were identified as major risk factors in our study.

Since the study aimed to evaluate genetic contributions to IS risk, genetic risk scores derived from the MetaGeno framework were included as independent variables in the model. Additionally, we tested the interaction between genetic risk and clinical comorbidities to examine potential gene-environment interactions. All survival analyses were conducted using the lifelines package in Python.

Concordance Index (C-Index)

The Concordance Index (C-index) was used to evaluate the discriminatory ability of our predictive model in ranking individuals based on their risk of developing IS. The C-index measures the probability that, for a randomly chosen pair of individuals, the individual with a higher predicted risk score experiences the event sooner. A C-index of 0.5 indicates a random prediction, whereas a value of 1.0 reflects perfect discrimination. The C-index is defined as:

$$C = \frac{\sum I(r_i > r_j)\delta_j}{\sum \delta_j}, \tag{3}$$

where r_i and r_j are the predicted risk scores for individuals i and j, $I(r_i > r_j)$ is an indicator function that takes the value 1 if the predicted risk score correctly ranks the two individuals, and δ_j is the event indicator (1 if the event occurred, 0 if censored).

We computed the C-index separately for each MRF to quantify its individual predictive power for IS risk. Additionally, we evaluated the C-index for combinations of diseases to assess the added value of integrating multiple MRFs in a multi-task learning framework. The C-index was estimated while accounting for censored observations, ensuring robustness in the presence of individuals without observed IS events during the study period.

To further validate the predictive performance, we computed bootstrapped confidence intervals (95% CI) for the C-index, resampling the dataset 1000 times to quantify the variability of the estimates. The statistical significance of differences between C-index values for different models was assessed using a paired concordance test.
